# Ketone Bodies as Metabolites and Signalling Molecules at the Crossroad between Inflammation and Epigenetic Control of Cardiometabolic Disorders

**DOI:** 10.3390/ijms232314564

**Published:** 2022-11-23

**Authors:** Nadia Bendridi, Anna Selmi, Aneta Balcerczyk, Luciano Pirola

**Affiliations:** 1 INSERM Unit 1060, CarMeN Laboratory, Faculty of Medicine, Lyon-1 University, 165 Chemin du Grand Revoyet-BP12, F-69495 Pierre-Benite, France; 2 Department of Molecular Biophysics, Faculty of Biology and Environmental Protection, University of Lodz, Pomorska 141/143, 90-236 Lodz, Poland

**Keywords:** ketone bodies, histone PTMs, histone β-hydroxybutyrylation, cardiovascular diseases

## Abstract

For many years, it has been clear that a Western diet rich in saturated fats and sugars promotes an inflammatory environment predisposing a person to chronic cardiometabolic diseases. In parallel, the emergence of ketogenic diets, deprived of carbohydrates and promoting the synthesis of ketone bodies imitating the metabolic effects of fasting, has been shown to provide a possible nutritional solution to alleviating diseases triggered by an inflammatory environment. The main ketone body, β-hydroxybutyrate (BHB), acts as an alternative fuel, and also as a substrate for a novel histone post-translational modification, β-hydroxybutyrylation. β-hydroxybutyrylation influences the state of chromatin architecture and promotes the transcription of multiple genes. BHB has also been shown to modulate inflammation in chronic diseases. In this review, we discuss, in the pathological context of cardiovascular risks, the current understanding of how ketone bodies, or a ketogenic diet, are able to modulate, trigger, or inhibit inflammation and how the epigenome and chromatin remodeling may be a key contributor.

## 1. Introduction

Ketone bodies constitute a family composed of three low-molecular-weight hydro-soluble molecules: acetone (Ac), β-hydroxybutyrate (BHB), and acetoacetate (AcAc), produced mainly by the liver [1] and to smaller extent by extrahepatic tissues including the intestinal crypts [2,3], kidneys [4], T cells [5], and astrocytes [6]. The capability of these tissues or cell lineages to produce ketone bodies depends on their mitochondrial expression of HMGCS2 (hydroxymethylglutaryl CoA synthase 2), the ketogenic rate-limiting enzyme [7,8] leading to ketone bodies production, using Ac-CoA and AcAc-CoA derived from the beta-oxidation of fatty acids. Ketogenesis and the use of ketone bodies as an alternative fuel is an adaptive metabolic mechanism, occurring in humans and mammals, that provides energy during periods of fasting, intense exercise, and when carbohydrates and/or glucose stores are limited [7]. Ketone bodies also play an essential role in infant brain development [9]. For many decades, the switch to a strictly ketogenic diet has been a clinically applied alternative to treating pharmacologically resistant epilepsy [10] and is a necessary approach in otherwise untreatable GLUT1-deficiency syndromes [11].

In addition, in recent years, the metabolism of ketone bodies has come under the spotlight for its possible beneficial effects on several metabolically related pathological conditions such as type 2 diabetes, obesity [12], and some types of cancers [13]. Two underlying mechanisms may be at work to confer such positive benefits to ketone bodies: their intrinsic anti-inflammatory properties and their capacity to induce a newly described epigenetic modification, namely histone β-hydroxybutyrylation [14], which may be mechanistically linked to the biological endpoint effects of ketone bodies.

In this review, we discuss the impact of ketone bodies on the physiology and pathological conditions of the organism by considering these molecules as epigenetic modifiers and from the perspective of their anti-inflammatory properties.

## 2. Biology of Ketone Bodies

### 2.1. Ketogenic and Ketolytic Metabolic Pathways

**Figure 1:**
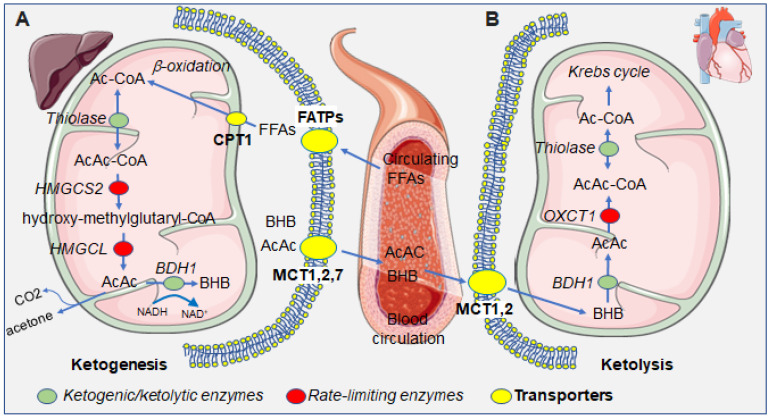
Summarizing scheme of the metabolism of ketone bodies in the hepatic and extrahepatic compartments. (**A**) In the liver, free fatty acids (FFAs) enter into the hepatocytes’s cytoplasm and then mitochondria, via fatty acid transporters (FATPs) and carnitine palmitoyltransferase I (CPT1), which serve as substrates for ketogenesis, leading to the production of the ketone bodies, BHB, AcAc, and the byproduct acetone. Ketone bodies that are exported from hepatocytes through the monocarboxylate transporters (MCTs) 1, 2, and 7. (**B**) In the heart, circulating ketone bodies enter cardiomyocytes through MCTs 1 and 2 and serve as substrates for ketolysis, ultimately yielding Ac-CoA which enters the Krebs cycle. Green dots represent enzymes taking part in both ketogenesis and ketolysis; red dots represent rate-limiting enzymes specific for either ketogenesis or ketolysis; yellow dots represent transporters, at the cellular or mitochondrial membrane. Abbreviations: Ac-CoA, acetyl coenzyme A; AcAc-CoA, acetoacetyl coenzyme A; AcAc, acetoacetate; BHB: β-hydroxybutyrate, FFAs, free fatty acids; *HMGCS2*: 3-hydroxy-3-methylglutaryl-CoA-synthase 2, *HMGCL*: hydroxymethylglutaryl-CoA lyase; *BDH1*, β-hydroxybutyrate dehydrogenase; *OXCT1*, 3-oxoacid CoA-transferase 1.

When lipolysis becomes predominant over lipogenesis, the rise in free fatty acids leads to an increase in the rate of conversion of Ac-CoA, the entry point for the Krebs cycle. Alternatively, and under conditions of carbohydrate deprivation, Ac-CoA serves as a ketogenic substrate. In the mitochondrial matrix, three enzymatic reactions convert two Ac-CoA molecules into acetoacetate, which is then reduced to form D-β-hydroxybutyrate. Firstly, an Acyl-CoA acetyltransferase (thiolase) condensates two molecules of Ac-CoA to produce AcAc-CoA. Subsequently, the enzyme HMG-CoA synthase (HMGCS2) adds another Ac-CoA group to form the intermediate β-hydroxymethylglutaryl-CoA (HMG-CoA). This second step is non-reversible and represents the rate-limiting enzymatic reaction of ketogenesis. Finally, HMG-CoA is converted to the ketone body acetoacetate by the enzyme HMG-CoA lyase. Acetoacetate can subsequently be reduced into the other, and quantitatively major, ketone body, β-hydroxybutyrate (BHB), by the enzyme β-hydroxybutyrate dehydrogenase (BDH1). Hepatically produced ketone bodies BHB and AcAc are released by the hepatocyte via the monocarboxylate transporter 1 (MCP1) and, through the circulation, reach extrahepatic tissues where they can be used as energy sources [15]. In the liver, AcAc and BHB levels reflect the redox balance of mitochondria [16]. Peripherally, BHB is reconverted into acetoacetate and then Ac-CoA, which enters the Krebs cycle. A minor fraction of acetoacetate is spontaneously decarboxylated into acetone that is for the most part exhaled by the breath [7]. When circulating ketone bodies concentrations are within the physiological limits, they act as energy substrates for multiple organs including the heart, brain, skeletal muscles, kidney, and mammary glands. It can be noted that erythrocytes, being devoid of mitochondria, cannot utilize ketone bodies as energetic substrates. Similarly, the liver, which does not have the enzyme *Oxct1*/SCOT1 to metabolize AcAc-CoA, is able to produce but not catabolize ketone bodies (Figure 1). Typically, ketone bodies are an alternative fuel under conditions of carbohydrate deprivation such as prolonged fasting or intense physical exercise. The metabolic switch towards ketogenesis is hormonally regulated by glucagon. Because of its occurrence in hepatic mitochondria, the metabolism of ketone bodies lies at the interface between the Krebs cycle, the β-oxidation of fatty acids, and sterol biosynthesis and is inversely correlated to lipogenesis. Unlike other metabolic pathways, such as glycolysis or the breakdown of fatty acids via β-oxidation that require energy consumption in some intermediate steps, the oxidation of ketone bodies is fully independent from ATP requirements [7].

### 2.2. Ketone Bodies as Epigenetic Modifiers

The metabolism of ketone bodies goes beyond the regulation of energy-providing metabolic pathways. Ketone bodies are also involved in the coordination of cellular functions through modification of the epigenome. In this context, histones, the protein constituents of the histone octamer and the nucleosome, play a fundamental role in modulating gene expression through their covalent post-translational modifications [17]. Histone methylation and acetylation of lysine residues have been widely studied as histone PTMs promoting transcription—such as histone acetylation at large [18] and histone methylation on the lysine 4 of histone H3—or acting as repressive marks such as methylation on the lysines 9 and 27 of histone H3 [19,20,21]. Numerous publications have already reported on the influence of ketosis on nuclear signalling in pathological contexts, low carbohydrate ketogenic diet, and the fasting state.

### 2.3. The Biochemical Basis of Histone β-Hydroxybutyrylation

In recent years, dietary strategies based on the provision of a ketogenic diet or ketone body precursors have attracted attention. The ketogenic diet may reduce blood glucose levels, promote insulin sensitivity, and reduce body weight both in mouse models [22] and in humans [23]. Indirectly, the ketogenic diet also ameliorates diabetes-related conditions such as nephropathy [24].

During exposure to a ketogenic diet, the organism switches to fat as a primary energy source, and the liver uses Ac-CoA derived from fatty acids to produce the ketone bodies β-hydroxybutyrate (BHB), acetoacetate, and acetone. In addition to the observed increase upon a KD, serum BHB levels are also augmented after prolonged exercise [25], fasting [26], and, under a pathological circumstance, diabetic ketoacidosis [27,28].

**Figure 2:**
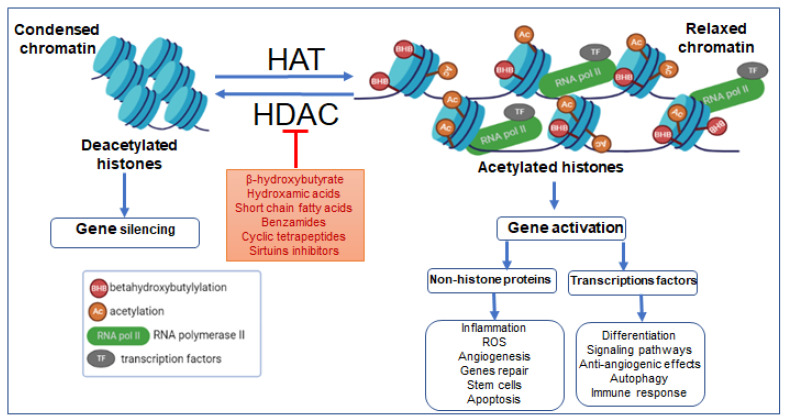
Effects of HAT and HDAC on β-hydroxybutyrylation, the chromatin’s transcriptional dynamics, and consequences of epigenetic remodeling on pathologies. Abbreviations: AC, acetylation; BHB, β-hydroxybutyrylation; TFs, transcription factors; RNA pol II, RNA polymerase II; ROS, reactive oxygen species.

BHB, within physiological levels, can attenuate the metabolic and cardiovascular manifestations of diabetes. BHB has been shown to reduce oxidative stress in streptozotocin-induced diabetic rats, preventing diabetic cardiomyopathy and cardiac death [29]. The molecular mechanisms linking BHB to epigenetic phenomena and diabetic physiopathology has only begun to be investigated in recent years. In a very elegant landmark study, histone β-hydroxybutyrylation (Kbhb) was identified by a mass spectrometry proteomic approach as a novel histone PTM in a mouse model of streptozotocin (STZ)-induced diabetes [14]. The original study reporting the discovery of β-hydroxybutyrylation also correlated this histone modification with transcriptional activity [14], suggesting that histone Kbhb, like acetylation and other histone acylations, participates in the determination of chromatin structure and regulation of gene expression. The enzymatic regulatory mechanisms governing β-hydroxybutyrylation have recently been elucidated. The histone acetyltransferase (HAT) p300 has been shown to be the major writer responsible for histone β-hydroxybutyrylation in vitro and in vivo and, in an in vitro reconstitution system of transcription using chromatin, p300 induced histone β-hydroxybutyrylation and p53 mediated transcriptional responses [30]. Removal of β-hydroxybutyrylation from histones is mediated by histone deacetylases (HDACs). Class I HDACs (HDAC1 and HDAC2) are zinc-dependent enzymes and class III HDACs (sirtuins: SIRT1, SIRT2) are NAD^+^-dependent enzymes, both removing acyl-modifications, and defined, by virtue of this activity, as erasers [31,32] (Figure 2). In the opposite way, another sirtuin isoform does not show β-hydroxybutyrylation action in in vitro assay using extracted core histones octamers as substrates [33]. On the other hand, a slow β-hydroxybutyrylation activity of SIRT3 has been shown on H3 peptides modified with both the S and R stereoisoforms of β-hydroxybutyrate, with a predominant activity towards the non-physiological S stereoisomer of the β-hydroxybutyryl group [34].

Physiological and pathological changes in BHB concentration under conditions such as starvation and diabetic ketoacidosis can cause Kbhb on many of histone’s lysine residues. Based on these findings, it is reasonable to assume that the KD also could cause these changes. In fact, in addition to indirectly participating in histone PTMs as a histone deacetylase (HDAC) inhibitor [35], a finding that is still debated [36], β-hydroxybutyrate can also directly regulate the complex process of gene transcription as an acyl donor, greatly expanding the epigenetic regulatory potential of BHB [35].

Additionally, the specific link between regulation of gene expression in diabetic complications and histone β-hydroxybutyrylation has been elucidated in recent studies. β-hydroxybutyrate treatment upregulated VEGF expression and promoted endothelial repair after vascular injury in diabetic rats [37]. During the repair process, BHB induced increased lysine β-hydroxybutyrylation and histone H3K9bhb in the aorta of diabetic rats [37]. Whether H3K9bhb is causally linked to the increased VEGF gene expression remains to be formally demonstrated, and in the affirmative, increased Kbhb may provide a putative intervention target to alleviate diabetic vascular dysfunction.

A recent study linked Kbhb to another biological response, with BHB upregulating the gene expression of MMP-2 by inducing specific β-hydroxybutyrylation in the MMP-2 promoter region in diabetic rats [38]. Such BHB-induced MMP-2 overexpression correlated with a reduction of the pathological changes of glomeruli and a decreased index of renal fibrosis [38]. Diabetic nephropathy is one of the major co-morbidities of diabetes, but effective treatments are still lacking. The above observations suggest a potential experimental basis for clinical translation of BHB use in treating diabetic nephropathy [39].

Further studies on Kbhb as a novel histone PTM will further elucidate its implication in epigenetic regulation and transcriptional activation phenomena. A proteomic analysis of mouse embryo fibroblasts identified 840 unique β-hydroxybutyrylation sites on 429 proteins. Lysine β-hydroxybutyrylation was found in enzymes belonging to the glycolytic and gluconeogenic pathways, pyruvate metabolism, and the Krebs cycle [40]. Additionally, a substantial portion of β-hydroxybutyrylated proteins is located in the nucleus, making histone β-hydroxybutyrylation a histone PTM that is overrepresented in the nucleus compared to other histone acylations such as butyrylation, succinylaction, and malonylation that are mainly non-nuclear [33]. In the liver, the proteomic analysis identified approximately 900 β-hydroxybutyrylation sites, mainly in genes involved in fatty acid and amino acid metabolism, detoxification pathways, and one-carbon metabolic pathways, suggesting that β-hydroxybutyrylation substantially alters the hepatic metabolism under ketogenic conditions [41]. Hence, Kbhb modifications, through modulation of gene expression of genes involved in metabolic pathways, may contribute to the development of metabolic dysfunction when dysregulated. This idea is consistent with a large amount of literature suggesting that BHB and β-hydroxybutyrylation are potentially protective against diabetes and diabetic complications.

### 2.4. BHB and Ketone Bodies as Signalling Mediators

G-protein-coupled receptors (GPCRs) are a highly versatile receptor family, responding to a large array of different ligands. A number of GPCRs are still defined as “orphans” —endogenous ligands that have not yet been identified [42]. Ketone bodies have been shown to be agonistic ligands for the GPCRs GPR81, GPR109A, and GPR109B, also defined as hydroxy-carboxylic acid 1, 2, and 3 receptors (HCARs), respectively [43]. As the expression of HCAR2 was also found in various immune cell lineages, including macrophages, it may be surmised that ketone bodies may play a role in the inhibition of inflammatory responses [44]. AS well as working as a GPCR activator, BHB has also been shown to be an antagonistic ligand for the GPR41, inducing a drop in intracellular cAMP and thus a decrease in lipolysis [45]. This decrease may allow the control of lipolysis during fasting periods, hence limiting excessive use of lipid stores [44]. In addition, a transcriptomic analysis revealed that BHB is also acting as in inhibitor of the MAPK signalling pathway, although the precise molecular target leading to such inhibition remains to be defined [46].

## 3. Ketone Bodies as an Alternative Fuel for the Heart

The heart is an extraordinarily flexible organ regarding its capability to adapt its metabolism to accommodate the use of different energy substrates for its functioning. The main sources of energy for the heart are fatty acids, glucose, lactate, certain amino acids, and ketone bodies. Their use depends greatly on their bioavailability as well as on the pathophysiological state of the individual [47].

The use of ketone bodies by the heart may be more beneficial than the use of glucose because no interim consumption of ATP is requested for the oxidation of ketone bodies, while some ATP is necessary to drive glycolysis. Ketone body catabolism by the heart depends on the capability of the liver to sustain ketogenesis using free fatty acids as substrates. Thereon, hepatically produced ketone bodies reach the bloodstream by crossing the mitochondrial and cytoplasmic membranes via MCTs (monocarboxylate transporters) [48]. Overall, there is a finely tuned balance between fatty acid and glucose metabolism in the heart. When oxidation of fatty acids is elevated, there is a reciprocal decrease in the oxidation of glucose. This close correlation, universally known as the Randle cycle after its proponent [49], is particularly impactful when the concentration of fatty acids becomes high and induces insulin resistance. In this case, insulin can no longer exert its inhibitory action on hepatic glucose production and on glucose uptake in peripheral tissues [50].

### 3.1. Cardiovascular Disease and Endothelial Damage Can Be Alleviated by Ketone Bodies

Diabetes, hypertension, abdominal obesity, and dyslipidemia are all metabolic disturbances that contribute to the appearance of atherosclerotic plaques and heart dysfunction. Hyperglycemia is a known promoter of endothelial dysfunction and an early contributor to the process of atherosclerosis [51]. The negative consequences of a hyperglycemic milieu on the endothelium are mediated by the production of free radicals (reactive oxygen species), activation of the PKC pathway, promotion of the hexosamine pathway, and an increase in advanced glycation products and polyols [52]. In addition to hyperglycemia, abdominal obesity also promotes the formation of atherosclerotic plaques. Pro-inflammatory adipocytokines released by the visceral adipose tissue, such as TNFα and plasminogen activator inhibitor-1 (PAI-1), cause damage to the vascular endothelium [53,54,55,56]. Concurrently, the increase in triglyceride levels and the decrease in HDL lead to a pro-atherosclerotic state [57]. Endothelial dysfunction linked to the development of atherosclerosis is also associated with the activation of the renin–angiotensin system [58], with angiotensin II leading to increased vasoconstriction, oxidative stress, inflammation, and thrombosis [59].

Insulin resistance is the third main contributor to damage of the vascular endothelium. As insulin inhibits lipolysis, in the insulin-resistant state, lipolysis is de-repressed and the vascular walls, exposed to higher levels of circulating fatty acids, will become more susceptible to apoptotic and inflammatory insults [60].

### 3.2. Potential Therapeutic Actions of the Ketogenic Diet

The ketogenic diet is a nutritional scheme low in carbohydrates and high in fat (between 70 and 80%) with a normal protein intake (10–20%). The very low carbohydrate content (5–10%) deprives the body of glucose as a primary energy source, rendering ketone bodies derived of fat as the main metabolic fuel. Extensive evidence suggests that ketogenic diets could, in several instances, play a therapeutic role. Medical practice has taken an interest in the ketogenic diet in the treatment of epilepsy, and this is now a standard of care for pharmacologically refractory epilepsies [61,62]. This therapeutic dietary approach was discovered at the Mayo Clinic in 1921 by Dr. Russell Wilder, who noticed that absolute fasting stopped epileptic seizures [63]. In an attempt to mimic the effects of fasting, leading to the production of ketone bodies but without massive loss of muscle mass, he pioneered the use of a ketogenic diet for pharmacologically refractory epilepsy.

The potential use of the ketogenic diet is now being investigated in other neurological conditions, including Alzheimer’s and Parkinson’s diseases, with preliminary promising results [64]. In the case of diabetes, both type II [65,66] and type I may be improved by adherence to a ketogenic diet, with caution to be exerted in type I diabetes due to the potential risk of hypoglycemia, diabetic ketoacidosis, and dyslipidemia [67]. Indeed, the concentration of ketone bodies must not exceed 15 mM, particularly in type I diabetics, otherwise metabolic acidosis will develop, leading to renal failure and cerebral edema [68]. An outpatient-clinic, small-scale study showed a marked improvement in diabetes in patients who observed a ketogenic diet, as the reduction of carbohydrate intake reduced insulin levels and promoted the use of ketone bodies [69].

### 3.3. The Role of Ketone Bodies in Inflammatory Disease

While on the one hand, inflammation is a body mechanism of defense against external pathogens and other insults, its dysregulation, leading to a temporary or permanent disproportionate reaction, leads to inflammatory diseases. One of the main molecular complexes mediating the inflammatory response is the NLRP3 inflammasome, a molecular complex including the proteins Nlrp3, ASC, and pro-caspase-1, which are expressed by different cell lineages such as macrophages, fibroblasts, and epithelial cells. The NLRP3 inflammasome is the best known inflammasome due to mutations on its exon 3 responsible for the appearance of serious inflammatory diseases including severe chronic infantile neurological cutaneous and articular syndrome; familial cold autoinflammatory syndrome (FCAS), also known familial cold urticaria; Muckle–Wells syndrome, and acute pancreatitis. Several factors can lead to the activation of the inflammasome including extracellular ATP, cholesterol crystals, uric acid crystals, hyaluronic acid, LPS, nucleic acids, and nanoparticles. Induction of oxidized mitochondrial DNA also activates the inflammasome [70]. A very recent clinical study showed that BHB could significantly reduce macrophage activation during acute pancreatitis [71]. Many studies converged on the idea that the presence of ketone bodies produced by the liver would reduce the mediators involved in inflammation and would constitute a promising treatment pathway for diabetes and autoimmune or neurodegenerative diseases [72]. In fact, BHB has been shown to inhibit the inflammatory complex NLRP3 through a decrease in intracellular potassium levels. In accordance, fasting for periods from 15 h up to two weeks, promoting the synthesis of ketone bodies, including BHB, would confer an anti-inflammatory status to the organism.

## 4. The Effects of a Ketogenic Diet on Inflammation-Dependent Atherosclerosis and Cardiovascular Risk

Atherosclerosis and cardiovascular risk are closely interrelated conditions. The formation of the atherosclerotic plaque is a slowly progressing event, with an initial accumulation of lipid deposits promoting inflammation, lesions of the vascular endothelium, and ultimately, narrowing and thickening of the vessels due to the formation of atheroma plaques [73]. Different evolutionary stages define the atherosclerotic progression—stages I, II, and III define early lesions, and stages IV, V, and VI define the more advanced lesions that induce the infiltration of macrophages into the intima mediated by the cytokine MCP-1/CCL2 [74], and deposition of extracellular lipids and cholesterol crystals [75].

In advanced stage V fibro-atheroma, the inflammasome is activated, with the secretion of IL-1β and IL-18 interleukins that generate an amplification loop leading to the activation of T lymphocytes and natural killer (NK) cells [76]. All these pro-inflammatory mechanisms mark the progression of atherosclerosis with inhibition of collagen synthesis which contributes to the resistance of the fibrous cap and the destabilization of the plaque, leading to the risk of plaque rupture and vascular ischemia [77]. Vascular inflammation is also accompanied by the activation of the biosynthetic pathway of prostaglandin E2 and the migration of macrophages in the arterial wall, whose cytokines exert pro-atherogenic effects. Subsequently, macrophage death leads to the release of their lipid content inducing a pro-thrombotic and necrotic core [78,79]. This latter event is an essential component in the instability of the plaques, their rupture, and the formation of intravascular clots [80].

### 4.1. The Effects of the Ketogenic Diet on the Inflammasome

Fat intake is traditionally viewed as a predisposing factor to weight gain and cardiovascular diseases. However, the ketogenic diet, via the induction of ketone body biosynthesis, could have a positive effect on the known risk factors for cardiovascular disease and atherosclerosis. It is widely accepted that the ingestion of omega-3 polyunsaturated fatty acids raises high-density lipoproteins (HDLs), also referred to as “good cholesterol”, and thus maintains a protective effect on the cardiovascular system. Studies have shown that a ketogenic diet has marked effects on blood triglyceride levels, lowering total cholesterol and raising HDL levels [81]. The ketogenic diet is thought to have significant effects on the size and volume of high-density lipoprotein particles that decrease cardiovascular risk compared to the smaller and denser low-density lipoprotein particles (LDLs) that increase cardiovascular risk [82]. Recent studies have shown that cardiovascular risk can be reduced by consuming a ketogenic diet [83]. The oxidation of ketone bodies as an energy source could counterbalance the oxidation of myocardial fatty acids, which are deleterious to the heart [84]. However, other studies have also shown that an excess of oxidation of ketone bodies would inhibit the functioning of the Krebs cycle in the myocardial cells of a perfused heart [85,86].

### 4.2. Targeting Cardiac Dysfunction with Ketone Bodies

**Figure 3:**
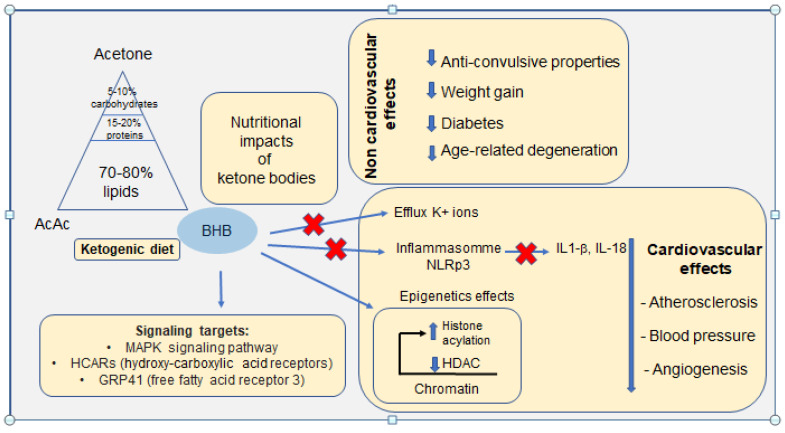
Schematic representation of the main links between the ketogenic diet on proven non-cardiovascular effects and physiological adaptations of the cardiovascular system. Recently identified signalling targets are also reported.

BHB has biochemical functions since it can inhibit several histone deacetylases [87,88], a family of nuclear proteins involved in gene regulation by eliminating an acetyl group present on the lysine residues of histones and non-histone proteins. This mechanism induces hyperacetylation of histones, thereby increasing accessibility to DNA transcriptional activity [89]. A recent study showed that in a mouse model of heart failure, upon administration of excess fatty acids, global hyperacetylation of mitochondrial proteins could be a major player driving inflammation and the assembly of NLPR3 [90]. Within this experimental model, the addition of BHB alleviated the mitochondrial dysfunctions induced by pro-inflammatory cytokines, thus preventing the deleterious effects of fatty acids on mitochondrial function. This study indicates that the direct reduction of mitochondrial protein acetylation by BHB, through activation of citrate synthase, could be an interesting therapeutic target for heart failure [91]. The use of BHB to inhibit inflammation can thus be an interesting treatment prospect since BHB is described as a ligand of GPR109A which inhibits lipolysis and exerts an anti-inflammatory effect [90]. Furthermore, a study by Yamanashi et al. (2017) [92] showed that BHB significantly reduced the activation of the NLRP3 complex in murine hippocampal cells. This inhibition was accompanied by a decrease in the synthesis of TNFα and Il-1β. These results support the hypothesis that BHB could constitute a molecule with wide-spectrum anti-inflammatory properties. The main links between the ketogenic diet and potential physiological adaptations are schematized in Figure 3.

## 5. Conclusions and Perspectives

The literature discussed in this review, representing a minor fraction of the abundant studies on the subject, provisionally supports the beneficial impact of physiological levels of ketone bodies, either produced by the organism during fasting or physical exercise or from a ketogenic diet, on cardiovascular pathologies. The main ketone body, BHB, may directly relieve conditions of oxidative stress and inflammation, exerting a beneficial effect on the causative factors of diseases such as diabetes and cardiometabolic disease [93]. Recent research has started to tackle the molecular mechanisms that come into play in the modulation of gene expression, transcriptionally promoting PTM modifications of histones upon administration of a ketogenic diet and/or ketone body precursors. Yet, questions still remain, or are only partially answered, about areas of public health that are of concern to all, such as: (i) How does fasting have an impact on cellular autophagy? (ii) Do ketone bodies affect DNA repair mechanisms? (iii) How is the ketogenic diet interrelated with the gut microbiome and the immune system? On the other hand, questions about the constraints imposed by a long-term ketogenic diet in terms of compliance and possible negative long-term effects should not be discounted. Finally, concerning the ketogenic diet, questions such as the nature of its constituent foods, the frequency of this diet, and the best period of life in which apply it to maximize its benefits on health and lifespan all remain open fields of investigation that will continue to grow and nurture scientific inquiry, to hopefully reach the wider applicative potential of ketone bodies or a KD as an adapted nutritional remedy to prevent, alleviate, control, or treat cardiometabolic disease in Western or other societies that adopt this nutritional model.

## Data Availability

Not applicable.

## Abbreviations

AcAc-CoA, acetoacetyl-coenzyme A; Ac-CoA, acetyl-coenzyme A; ASC, apoptosis-associated speck-like protein; ATP, adenosine triphosphate; BHB, hydroxybutyrate; CCL2, chemokine ligand 2; GLUT1, glucose transporter 1; FATPs, fatty acids transport proteins; HDAC, histone deacetylase; HAT, histone acetyltransferase; HDLs, high density lipoproteins; HMGCS2, 3-hydroxy-3-methylglutaryl-CoA synthase 2; Kbhb, β-hydroxybutyrylation; KD, ketogenic diet; LDLs, low-density lipoproteins; MCP1, monocyte chemoattractant protein 1; MCTs, monocarboxylate transporters; MMP-2, matrix metalloproteinase-2; NK, natural killer; NLRP3, NOD-like receptor family, pyrin domain containing 3; Oxct1, 3-oxoacid CoA-transferase; PAI-1, plasminogen activator inhibitor-1; PKC, protein kinase C; PTMs, post-translational modifications; SCOT1, succinyl-CoA,3-ketoacid CoA transferase; TNFα, tumor necrosis factor α.
